# Ferroptosis-Related GPX4 and CD71 and Immune Checkpoint PD-L1 in Cutaneous Melanoma: Associations with Clinicopathologic Features and Survival Outcomes

**DOI:** 10.3390/ijms27209278

**Published:** 2026-10-19

**Authors:** Rukiye Yasak Güner, Ramazan Oğuz Yüceer, Ahmet Turan Ünsal, Melih Akyol, Hatice Reyhan Eğilmez

**Affiliations:** 1 Department of Dermatology, Faculty of Medicine, Sivas Cumhuriyet University, 58140 Sivas, Turkey; rky.guner@gmail.com (R.Y.G.); melakyoll@gmail.com (M.A.); 2 Department of Pathology, Faculty of Medicine, Sivas Cumhuriyet University, 58140 Sivas, Turkey; regilmez@cumhuriyet.edu.tr; 3 Department of Dermatology, Artvin State Hospital, 08000 Artvin, Turkey; ahmeturanunsal@gmail.com

**Keywords:** cutaneous melanoma, ferroptosis, glutathione peroxidase 4 (GPX4), transferrin receptor 1 (CD71), programmed death-ligand 1 (PD-L1), biomarkers, prognosis

## Abstract

Ferroptosis-related pathways and immune checkpoint signaling may contribute to melanoma progression, but their clinicopathologic and prognostic interplay remains incompletely characterized. To investigate GPX4, CD71, and PD-L1 expression in primary cutaneous melanoma and evaluate their associations with clinicopathologic features and survival outcomes. This retrospective single-center cohort included 44 patients with primary cutaneous melanoma. PD-L1, GPX4, and CD71 expressions were assessed immunohistochemically. Associations with clinicopathologic parameters were analyzed, and overall survival (OS) and disease-free survival (DFS) were evaluated using Kaplan–Meier and Cox regression analyses. PD-L1, GPX4, and CD71 positivity was were observed in 25.0%, 38.6%, and 36.4% of tumors, respectively. PD-L1 expression was associated with Breslow thickness (*p* = 0.004), whereas CD71 expression was associated with perineural invasion, Clark level, and disease stage (*p* = 0.019, *p* = 0.034, and *p* = 0.045, respectively). GPX4-positive tumors showed significantly shorter OS (40 vs. 103 months; *p* = 0.003) and DFS (42 vs. 86 months; *p* = 0.009). Triple-positive tumors (PD-L1^+^/GPX4^+^/CD71^+^) demonstrated significantly shorter OS and DFS than triple-negative tumors (*p* = 0.035 and *p* = 0.040, respectively). In multivariable analysis, GPX4 positivity remained independently associated with poorer OS. GPX4 expression was associated with adverse survival outcomes in cutaneous melanoma, while combined GPX4, CD71, and PD-L1 expression identified a subgroup with particularly unfavorable outcomes. These findings support further investigation of ferroptosis-related markers alongside immune checkpoint pathways as candidate prognostic biomarkers in melanoma.

## 1. Introduction

Cutaneous melanoma is among the most aggressive skin malignancies, with a strong tendency for metastasis and a substantial contribution to skin cancer-related mortality. The clinical course is highly variable, ranging from localized tumors that can be successfully treated with surgery to rapidly progressive disease with regional lymph node and distant organ involvement. Breslow thickness, ulceration, mitotic activity, and lymph node status remain important parameters for staging and prognostic assessment [1]. However, patients with similar clinicopathologic features and disease stages may experience considerably different outcomes, highlighting the biological heterogeneity of melanoma. Despite major advances in treatment with immune checkpoint inhibitors and targeted therapies, recurrence, disease progression, and treatment resistance remain important clinical problems [2]. Therefore, there is continued interest in identifying tissue biomarkers that may complement established clinicopathologic parameters and improve our understanding of melanoma behavior.

Melanoma is an immunogenic tumor, and its interaction with the local immune microenvironment has an important influence on tumor progression and treatment response. Programmed death-ligand 1 (PD-L1) is one of the main molecules involved in this interaction. Through binding to programmed death-1 (PD-1), PD-L1 suppresses T-cell activity and contributes to tumor immune escape [3]. Consequently, the PD-1/PD-L1 pathway has become an important therapeutic target in advanced melanoma. However, the clinical significance of PD-L1 expression itself is unclear. Studies evaluating its prognostic role have reported inconsistent findings, and responses to immune checkpoint inhibitors have also been observed in patients with PD-L1–negative tumors [4,5]. Therefore, PD-L1 expression alone appears insufficient to explain the complex relationship between melanoma cells, antitumor immunity, and clinical outcomes [6]. The evaluation of PD-L1 together with other mechanisms involved in tumor cell survival may provide additional biological information.

Ferroptosis has recently attracted attention as a mechanism. It is a regulated form of cell death driven by iron-dependent lipid peroxidation and differs mechanistically from apoptosis and other forms of cell death [7]. Melanoma cells are exposed to considerable metabolic and oxidative stress, making the regulation of ferroptosis potentially important for their survival. Experimental studies have shown that melanoma cells differ in their susceptibility to ferroptosis according to their differentiation and cellular states. In particular, treatment-resistant cell populations may acquire mechanisms that allow them to escape ferroptotic cell death, potentially contributing to tumor persistence and progression [8,9].

Glutathione peroxidase 4 (GPX4) is a major regulator of this process. GPX4 reduces lipid hydroperoxides and thereby protects cellular membranes from lipid peroxidation, which leads to ferroptosis. This antioxidant activity may become particularly important for tumor cells exposed to high levels of oxidative stress [10]. Experimental studies have shown that persistent and treatment-resistant cancer cells can become dependent on GPX4 for survival. Such a mechanism may have particular relevance in melanoma, where cellular plasticity and adaptation to therapeutic pressure are recognized features of disease progression and treatment resistance [11,12,13].

Iron availability is also critical for ferroptosis. Transferrin receptor 1 (CD71) is responsible for the cellular uptake of transferrin-bound iron and is commonly expressed at higher levels in metabolically active and proliferating cells [14]. Iron supports several processes required for tumor growth, while excess intracellular iron can increase reactive oxygen species and promote lipid peroxidation. Therefore, CD71 links cellular iron metabolism with both tumor proliferation and ferroptotic susceptibility. Increased CD71 expression has been associated with aggressive tumor characteristics in several malignancies, although its clinical significance in cutaneous melanoma has not been well established [15,16].

There is also growing evidence that ferroptosis and antitumor immunity are closely interconnected. Activated CD8+ T cells can increase lipid peroxidation and promote ferroptosis in tumor cells, while ferroptotic cell death itself can modify the immune microenvironment [17]. At the same time, mechanisms that limit lipid peroxidation, including GPX4 activity, may help tumor cells survive immune-mediated oxidative stress. These findings suggest a biological link between immune escape, iron metabolism, and ferroptosis that may be especially relevant in melanoma, where immune-based treatment has become a central component of clinical management [18].

PD-L1, GPX4, and CD71 represent different but interconnected aspects of melanoma biology: immune escape, protection against ferroptotic cell death, and cellular iron metabolism, respectively. Examining these markers together may therefore provide a broader view of the biological processes associated with aggressive tumor behavior. However, their expression and clinical significance have rarely been evaluated together within the same cutaneous melanoma cohort. In this study, we evaluated the immunohistochemical expression of PD-L1, GPX4, and CD71 and examined their associations with clinicopathologic characteristics, disease-free survival, and overall survival. We also assessed whether their combined evaluation could provide additional prognostic information in patients with cutaneous melanoma.

## 2. Results

The study included 44 patients with cutaneous melanoma, of whom 26 (59.1%) were male and 41 (93.2%) were aged ≥45 years. Superficial spreading melanoma was the most common histologic subtype (47.7%), followed by nodular melanoma (41.0%). Ulceration and lymphovascular invasion were present in 43.2% and 27.3% of cases, respectively. Breslow thickness was >4.0 mm in 36.4% of patients, and 45.5% had stage III disease. Lymph node involvement was identified in 45.5% of patients, whereas the BRAF V600E mutation was detected in 18.2% (Table 1).

PD-L1 positivity was observed in 11 (25.0%) of 44 tumors. PD-L1 expression was significantly associated with comorbidity status (*p* = 0.007) and Breslow thickness (*p* = 0.004). TILs were more frequent in PD-L1-positive than PD-L1-negative tumors (45.5% vs. 18.2%), although this difference did not reach statistical significance (*p* = 0.083). No significant associations were observed with the other clinicopathologic parameters (*p* > 0.05).

CD71 positivity was detected in 16 (36.4%) tumors and was significantly associated with perineural invasion (*p* = 0.019), Clark level (*p* = 0.034), and tumor stage (*p* = 0.045). Perineural invasion was more frequent in CD71-positive than in CD71-negative tumors (37.5% vs. 7.1%), and CD71-positive tumors more frequently showed Clark level IV (56.3% vs. 32.1%) and stage III disease (56.3% vs. 39.3%). Lymphovascular invasion was also more frequent in CD71-positive tumors (43.8% vs. 17.9%), although the association did not reach statistical significance (*p* = 0.068). No significant associations were found with the remaining variables.

**Figure 1:**
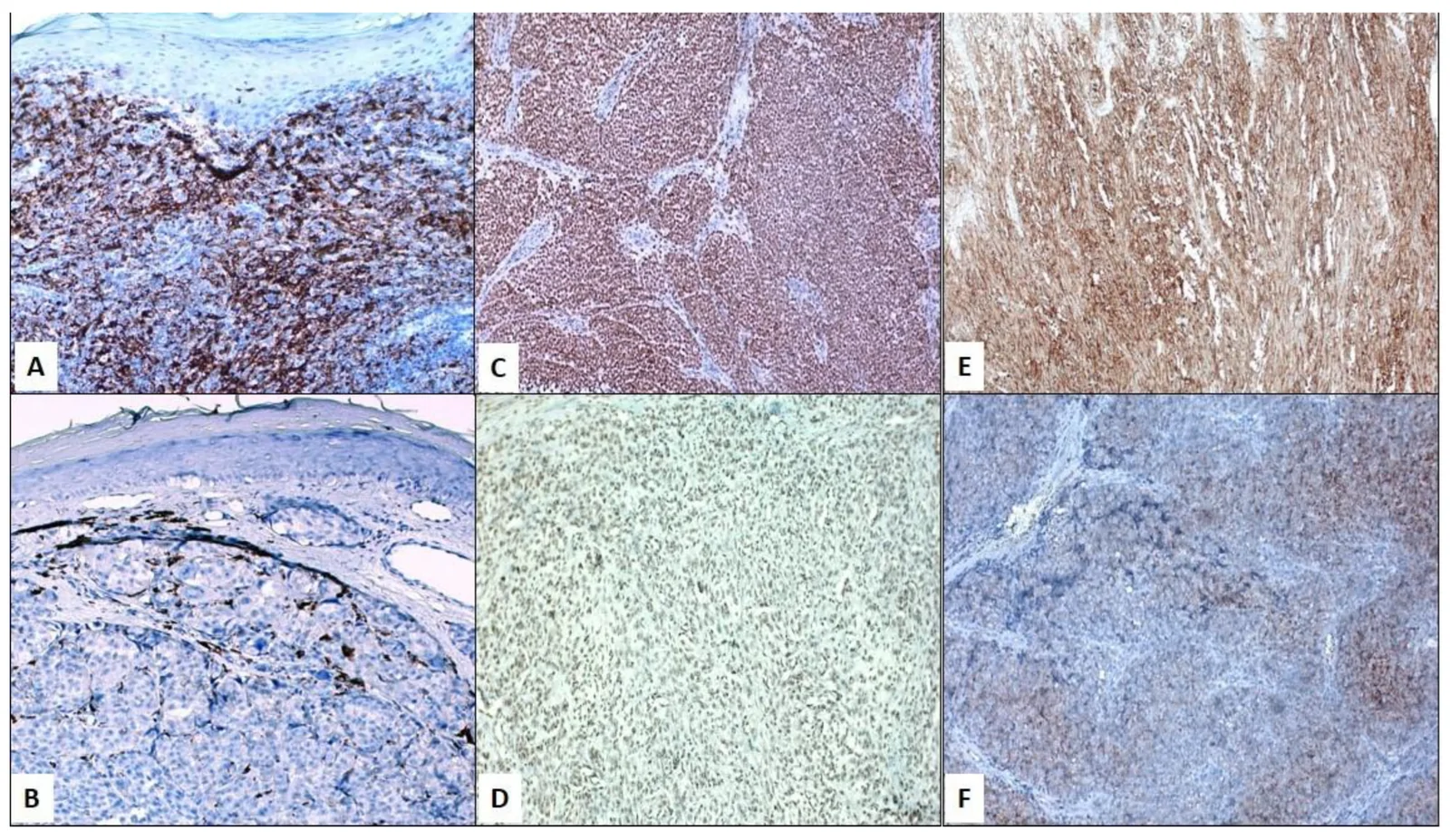
Representative immunohistochemical expression patterns of PD-L1, GPX4, and CD71 in primary cutaneous melanoma. Representative tumor sections demonstrating (**A**) positive PD-L1 immunoreactivity and (**B**) negative PD-L1 expression (original magnification, ×100); (**C**) positive GPX4 immunoreactivity and (**D**) negative GPX4 expression (original magnification, ×200); and (**E**) positive CD71 immunoreactivity and (**F**) negative CD71 expression (original magnification, ×200). PD-L1 positivity was characterized by membranous staining of tumor cells, GPX4 positivity by cytoplasmic staining, and CD71 positivity by membranous and/or cytoplasmic staining.

GPX4 positivity was detected in 17 (38.6%) tumors. GPX4 expression was not significantly associated with any clinicopathologic parameter (*p* > 0.05). However, GPX4-positive tumors showed higher frequencies of perineural invasion (29.4% vs. 11.1%) and lymphovascular invasion (41.2% vs. 18.5%), although neither association reached statistical significance (*p* = 0.130 and *p* = 0.098, respectively). Male sex (*p* = 0.060) and family history (*p* = 0.065) also showed borderline associations with GPX4 expression (Figure 1, Table 2). GPX4-positive tumors showed higher frequencies of PNI and LVI than GPX4-negative tumors. The odds of PNI were approximately threefold higher in GPX4-positive tumors (OR 3.33, 95% CI 0.68–16.35; *p* = 0.227), as were the odds of LVI (OR 3.08, 95% CI 0.78–12.12; *p* = 0.164). However, neither association reached statistical significance.

**Figure 2:**
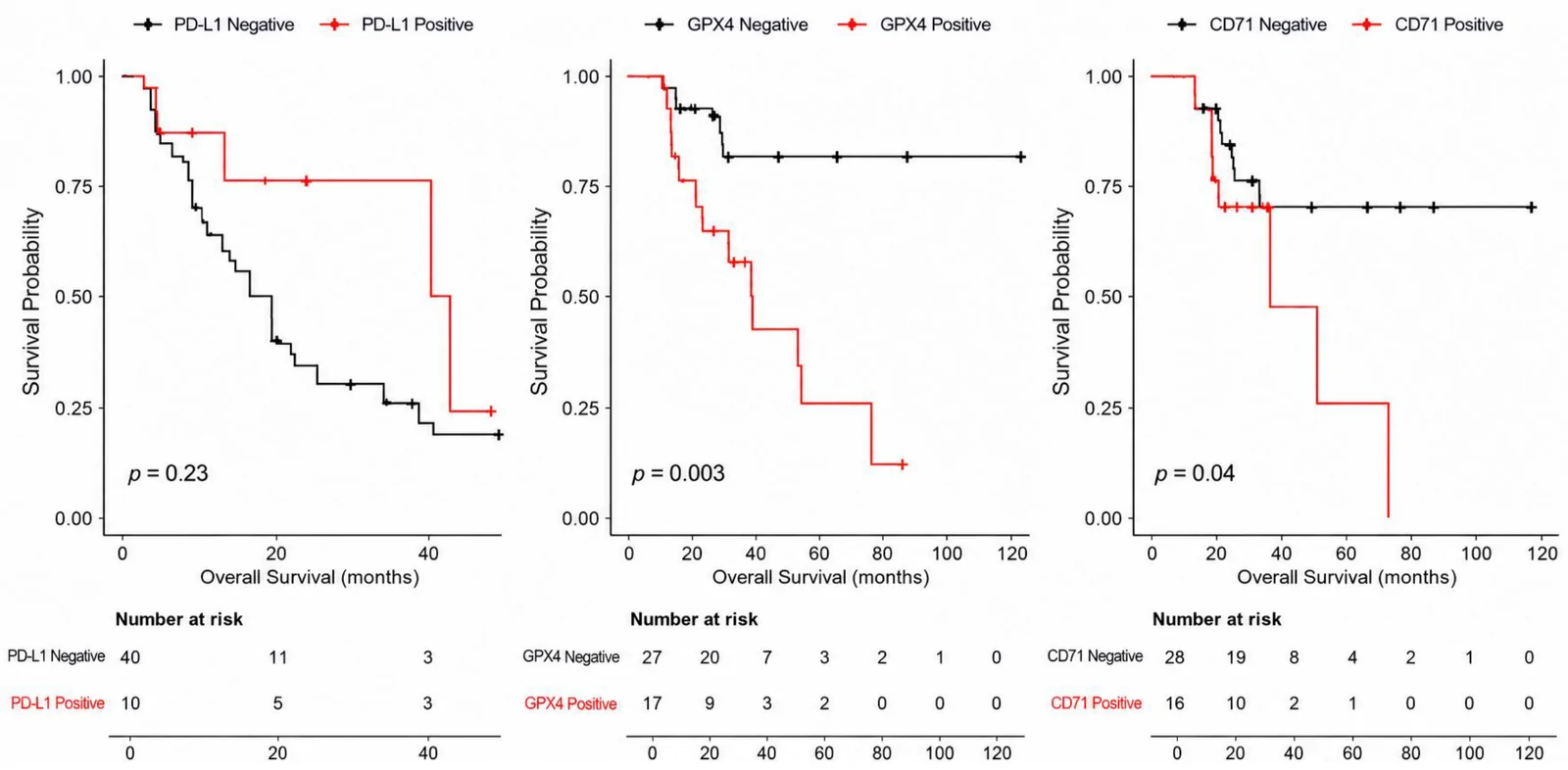
Kaplan–Meier overall survival (OS) curves according to PD-L1, GPX4, and CD71 expression status in melanoma patients.

During a median follow-up of 78.9 months, 12 patients (27.3%) died, and the median overall survival (OS) for the entire cohort was 76 months (95% CI, 56.5–95.7). Median OS was shorter in PD-L1-positive than in PD-L1-negative patients (42 vs. 90 months), although the difference was not statistically significant (*p* = 0.230). In contrast, GPX4-positive patients had significantly shorter median OS than GPX4-negative patients (40 vs. 103 months; *p* = 0.003). Similarly, CD71 positivity was associated with significantly shorter OS (40 vs. 93 months; *p* = 0.040) (Figure 2).

**Figure 3:**
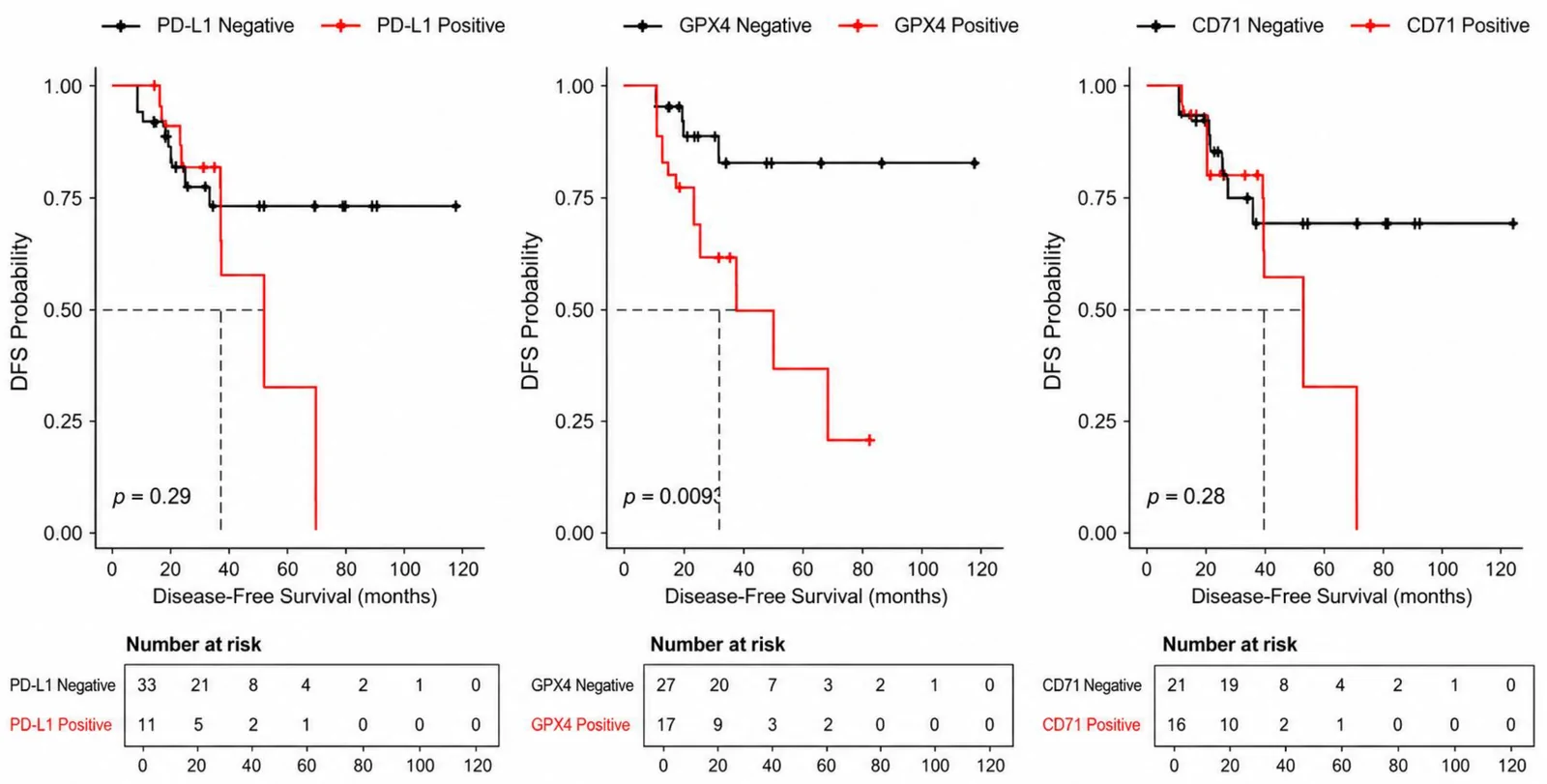
Kaplan–Meier Disease-Free Survival (DFS) curves according to PD-L1, GPX4, and CD71 expression status in melanoma patients.

During follow-up, recurrence occurred in 13 patients (29.5%), and the median disease-free survival (DFS) for the overall cohort was 74 months (95% CI, 54.9–93.8). PD-L1 expression was not significantly associated with DFS (*p* = 0.296). GPX4-positive patients with GPX4 expression had significantly shorter median DFS than those without GPX4 (42 vs. 86 months; *p* = 0.009). Although CD71-positive patients also had shorter median DFS than those without CD71 (40 vs. 99 months), this difference did not reach statistical significance (*p* = 0.280) (Figure 3).

Triple-negative (PD-L1^−^/CD71^−^/GPX4^−^) and triple-positive (PD-L1^+^/CD71^+^/GPX4^+^) groups were compared for overall survival (OS). Death occurred in four of 18 patients (22.2%) in the triple-negative group and four of five patients (80.0%) in the triple-positive group. Kaplan–Meier analysis demonstrated significantly shorter OS in the triple-positive group (log-rank *p* = 0.035).

**Figure 4:**
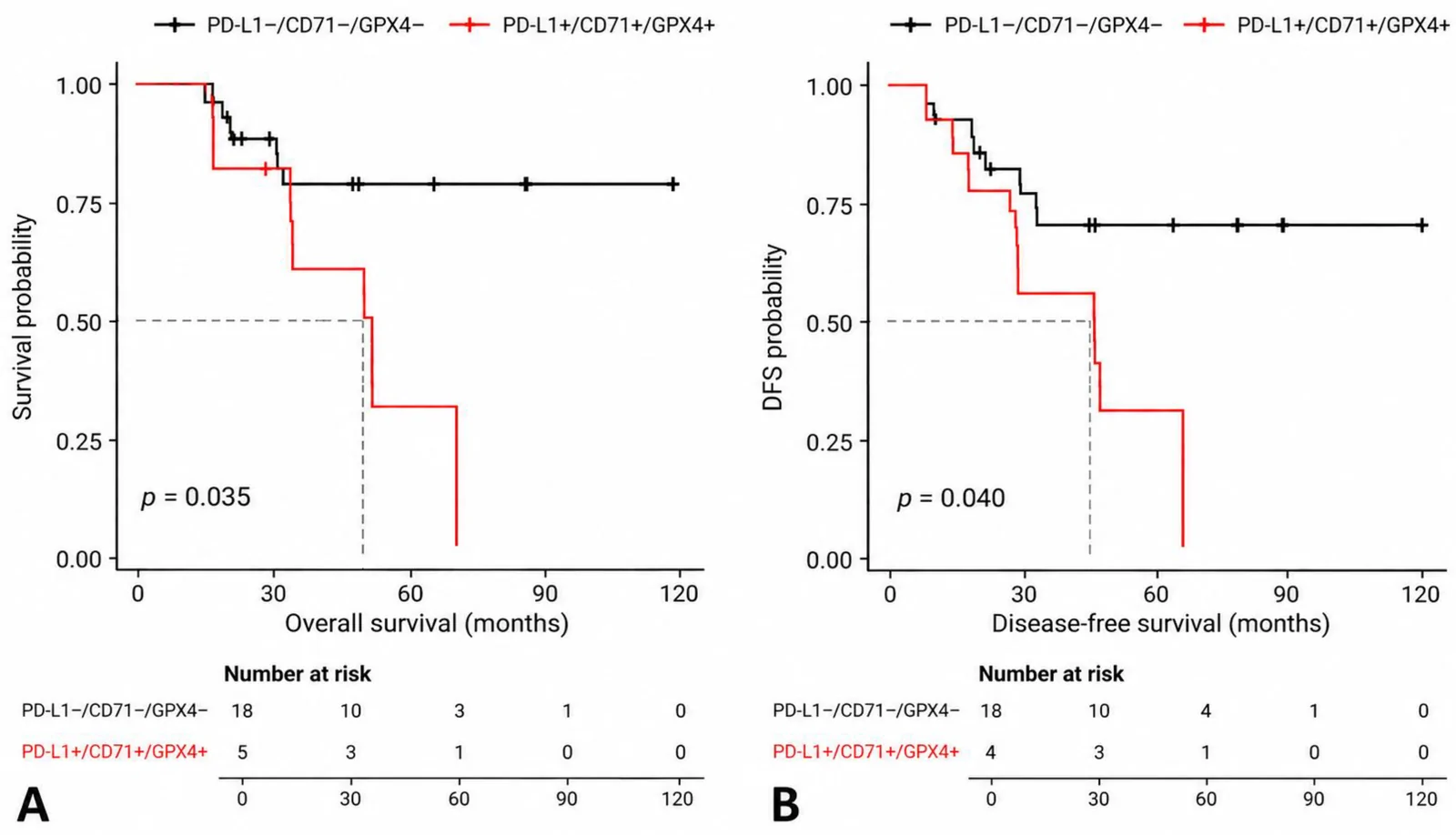
Kaplan–Meier survival curves according to combined PD-L1, CD71, and GPX4 expression status. (**A**) Overall survival (OS) comparison between triple-negative and triple-positive groups (*p* = 0.035); (**B**) Disease-free survival (DFS) comparison between triple-negative and triple-positive groups (*p* = 0.040).

Disease-free survival (DFS) was also compared between the triple-negative and triple-positive groups. Recurrence occurred in four of 18 patients (22.2%) in the triple-negative group and in four of five patients (80.0%) in the triple-positive group. Kaplan–Meier analysis showed significantly shorter DFS in the triple-positive group (log-rank *p* = 0.040) (Figure 4).

In univariable Cox regression analysis for overall survival (OS), lymphovascular invasion (LVI) and GPX4 expression were significant prognostic factors. LVI was associated with an approximately 3.3-fold increased risk of death (HR, 3.302; 95% CI, 1.058–10.305; *p* = 0.040), whereas GPX4 positivity was associated with an approximately 5.8-fold increased risk (HR, 5.781; 95% CI, 1.559–21.434; *p* = 0.009). Sex (HR, 3.957; 95% CI, 0.854–18.337; *p* = 0.079) and CD71 expression (HR, 3.178; 95% CI, 0.996–10.138; *p* = 0.051) showed borderline associations with OS. No significant associations were observed for the remaining clinicopathologic variables (all *p* > 0.05).

For disease-free survival (DFS), LVI and GPX4 expression were significantly associated with the outcome. LVI was associated with an approximately 4.1-fold increased risk of recurrence (HR, 4.086; 95% CI, 1.368–12.201; *p* = 0.012), and GPX4 positivity with an approximately 4.2-fold increased risk (HR, 4.221; 95% CI, 1.295–13.760; *p* = 0.017). Sex showed a borderline association with DFS (HR, 4.495; 95% CI, 0.985–20.510; *p* = 0.052), whereas no other clinicopathologic variables were significantly associated with DFS (*p* > 0.05) (Table 3).

In the multivariable Cox regression analysis for overall survival (OS), GPX4 expression remained an independent prognostic factor in Model 1 (HR, 4.845; 95% CI, 1.278–18.369; *p* = 0.020), whereas lymphovascular invasion (LVI) was not significant (HR, 2.402; 95% CI, 0.739–7.809; *p* = 0.145). After inclusion of CD71 expression in Model 2, GPX4 retained its independent prognostic significance (HR, 4.333; 95% CI, 1.022–18.368; *p* = 0.047), whereas LVI and CD71 were not significantly associated with OS (*p* = 0.254 and *p* = 0.684, respectively).

For disease-free survival (DFS), LVI was an independent prognostic factor in Model 1 (HR, 3.172; 95% CI, 1.025–9.810; *p* = 0.045), whereas GPX4 showed a borderline association (HR, 3.335; 95% CI, 0.997–11.164; *p* = 0.051). After adjustment for sex in Model 2, LVI remained independently associated with DFS (HR, 3.911; 95% CI, 1.174–13.031; *p* = 0.026), whereas GPX4 and sex were not significant (*p* = 0.424 and *p* = 0.145, respectively) (Table 4).

## 3. Discussion

Melanoma is a heterogeneous malignancy in which immunologic and metabolic alterations, as well as mechanisms of treatment resistance, influence tumor biology. In recent years, particularly with the introduction of immune checkpoint inhibitors into clinical practice, there has been increasing interest in identifying novel biomarkers that may predict treatment response and prognosis [19]. A better understanding of the relationship between ferroptosis and tumor immunity has further increased interest in biomarkers linking immune regulation with cellular iron metabolism. In the present study, PD-L1, CD71, and GPX4 expression were evaluated concurrently in the same cohort of patients with cutaneous melanoma, and their associations with clinicopathologic characteristics and survival were investigated. Our findings showed that GPX4 expression was significantly associated with poorer overall survival after adjustment in parsimonious multivariable Cox models, whereas CD71 expression was associated with more aggressive tumor features and poorer clinical outcomes. In contrast, PD-L1 expression alone did not demonstrate significant prognostic value. Moreover, patients with concurrent positivity for all three biomarkers had shorter overall and disease-free survival, suggesting that biomarker combinations reflecting both immune escape and ferroptosis resistance may provide additional prognostic information. However, given the small number of triple-positive cases, this finding should be considered preliminary and hypothesis-generating.

The prognostic significance of PD-L1 in melanoma remains controversial. In our study, PD-L1 expression was not significantly associated with overall or disease-free survival, consistent with previous reports suggesting that although PD-L1 is an important predictive biomarker in the context of immunotherapy, its value as an independent prognostic marker may be limited [20]. PD-L1 expression was significantly associated with Breslow thickness; however, PD-L1 positivity did not show a clear progressive increase across increasing Breslow thickness categories. Therefore, this association should not be interpreted as evidence of a direct relationship between increasing tumor thickness and increasing PD-L1 expression. The higher frequency of TILs among PD-L1-positive tumors may suggest an association between PD-L1 expression and the immune tumor microenvironment, although this finding also requires confirmation in larger cohorts [21].

CD71 expression has been associated with aggressive biological behavior and poor prognosis in several solid tumors [22]. In our cohort, CD71 positivity was associated with perineural invasion, higher Clark level, and more advanced tumor stage, supporting a potential relationship between CD71 expression and a more invasive melanoma phenotype. CD71-positive patients also had shorter overall and disease-free survival. However, CD71 did not retain independent prognostic significance in multivariable analysis, suggesting that its prognostic effect may be related, at least in part, to its association with invasive tumor characteristics rather than representing an independent effect [23]. Although CD71 is involved in cellular iron uptake and is closely related to pathways relevant to ferroptosis, its immunohistochemical expression alone cannot be considered a direct measure of ferroptotic activity [24].

Experimental studies have demonstrated that GPX4-mediated ferroptosis resistance is involved in melanoma progression and treatment resistance [25]. In our study, GPX4 expression was not significantly associated with individual clinicopathologic characteristics but was significantly associated with poorer overall survival after adjustment in parsimonious multivariable Cox models. However, increased GPX4 immunohistochemical expression alone does not demonstrate ferroptosis resistance or provide a functional measure of ferroptotic activity. Therefore, our findings should be interpreted as an association between GPX4 expression and clinical outcome rather than direct evidence of ferroptosis resistance in melanoma. Functional studies incorporating additional measures of lipid peroxidation, oxidative stress, and ferroptotic activity are needed to clarify the biological basis of this association.

Another notable finding was the significantly shorter overall and disease-free survival among patients with concurrent PD-L1, CD71, and GPX4 positivity (PD-L1^+^/CD71^+^/GPX4^+^). Although these markers are involved in biological pathways related to immune regulation, iron metabolism, and ferroptosis, their concurrent immunohistochemical expression does not establish functional interaction between these pathways. Moreover, only five patients had the triple-positive phenotype, and patients with mixed-expression profiles were not included in this comparison. Although patients with the triple-positive phenotype had poorer OS and DFS than those with the triple-negative phenotype, it remains unclear whether this combination offers prognostic information beyond GPX4 alone. The small number of triple-positive cases and outcome events did not allow a reliable multivariable comparison of the combined phenotype with GPX4 expression alone. Thus, these findings should be interpreted with caution and regarded as exploratory. Further studies in larger cohorts are needed to determine whether the combined assessment of PD-L1, CD71, and GPX4 improves prognostic stratification over individual biomarkers. Previous experimental studies have demonstrated close interactions between ferroptosis and antitumor immunity and have suggested potential links between GPX4- and CD71-related metabolic pathways and PD-L1-mediated immune regulation [22,26,27]. Our results cannot establish a direct mechanistic relationship between these pathways; nevertheless, the poorer outcomes observed in triple-positive tumors provide a clinical rationale for investigating them together rather than as isolated biomarkers. Preclinical evidence showing enhanced antitumor activity when ferroptosis-inducing strategies are combined with immune checkpoint inhibition further highlights the potential relevance of this interaction. Larger studies are needed to determine whether the PD-L1/GPX4/CD71 profile could contribute to prognostic stratification or help identify patients who may benefit from therapeutic approaches targeting both ferroptosis and immune checkpoint pathways.

A strength of this study is the simultaneous evaluation of PD-L1, CD71, and GPX4 within the same cutaneous melanoma cohort, supported by comprehensive clinicopathologic characterization and long-term follow-up. Nevertheless, several limitations should be considered. The retrospective, single-center design and relatively small sample size may have introduced selection bias and limited statistical power, particularly in subgroup and multivariable analyses. The small number of triple-positive cases requires cautious interpretation of the survival findings. In addition, multiple clinicopathologic associations and survival comparisons were examined without adjustment for multiple testing because of the exploratory nature of the study. Therefore, the possibility of type I error and false-positive findings cannot be excluded, particularly for marginally significant associations, and these results should be considered hypothesis-generating and interpreted cautiously. Immunohistochemical assessment may also be influenced by intratumoral heterogeneity and differences in scoring methods. Treatment-related variables were not comprehensively included in the survival models and may represent potential confounders. Finally, the absence of an independent validation cohort limits generalizability. Treatment-related factors, including immunotherapy and targeted therapy, were not fully accounted for in the survival analyses and may have influenced the observed outcomes. Larger multicenter studies with standardized biomarker assessment, appropriate control of multiple comparisons, and independent validation are needed to confirm these findings.

## 4. Materials and Methods

### 4.1. Study Design and Patient Selection

This retrospective, single-center cohort study included patients diagnosed with melanoma at the Sivas Cumhuriyet University Department of Dermatology and Pathology between 1 January 2015 and 1 December 2025. Pathology archives and electronic medical records were retrospectively reviewed. A total of 60 patients were initially screened. Eligibility criteria included histologically confirmed primary cutaneous melanoma, availability of adequate formalin-fixed paraffin-embedded (FFPE) tumor tissue for immunohistochemical analysis, and sufficient clinicopathologic and follow-up data.

Of the 60 patients screened, 16 were excluded because of noncutaneous melanoma, including mucosal and ocular melanoma (*n* = 5); insufficient or extensively necrotic tumor tissue or unavailable paraffin blocks (*n* = 6); or inadequate clinical or follow-up data (*n* = 5). The final study cohort comprised 44 patients with primary cutaneous melanoma.

Demographic, clinicopathologic, molecular, and follow-up data were obtained from pathology reports and institutional medical records. Variables included age, sex, comorbidities, family history, anatomic site, histologic subtype, Breslow thickness, Clark level, ulceration, neurotropism, perineural and lymphovascular invasion, microsatellitosis, tumor-infiltrating lymphocytes (TILs), regional lymph node status, disease stage, and BRAF V600E mutation status. Tumor-infiltrating lymphocytes (TILs) were evaluated on hematoxylin and eosin-stained sections by assessing lymphocytic infiltration within and around the invasive tumor. Cases showing lymphocytic infiltration associated with the tumor were classified as TIL-positive, whereas cases without identifiable tumor-associated lymphocytic infiltration were classified as TIL-negative. BRAF V600E mutation status was assessed by PCR using DNA extracted from representative FFPE tumor tissue. Tumors with a detected V600E mutation were considered BRAF V600E-positive, while those without the mutation were considered negative.

**Figure 5:**
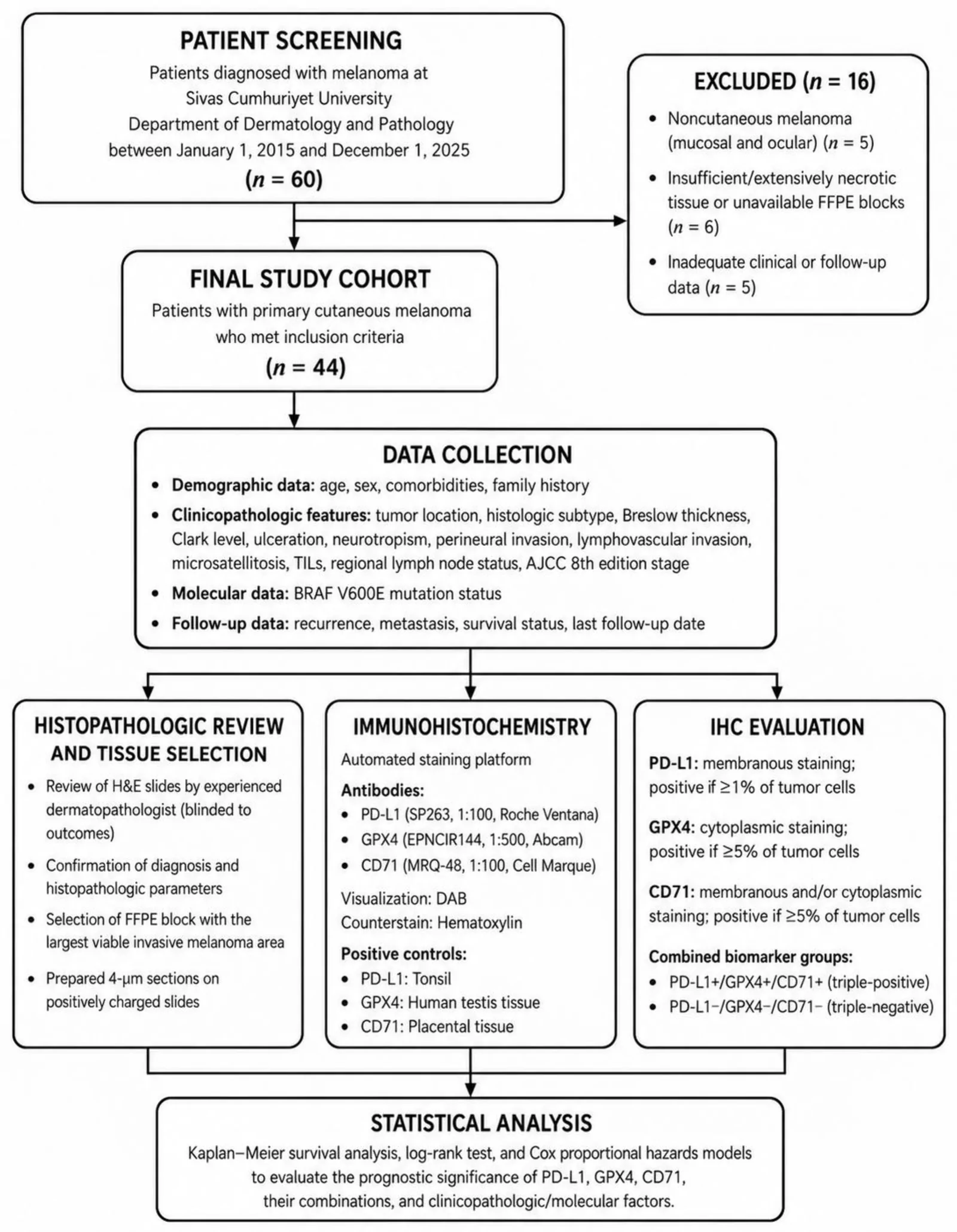
Study flowchart.

Tumor location was categorized as head and neck, trunk, upper extremity, or lower extremity. Histologic subtypes were classified as superficial spreading, nodular, or lentigo maligna melanoma. Breslow thickness was categorized as ≤1.0, >1.0–2.0, >2.0–4.0, or >4.0 mm. Histopathologic parameters were reassessed using the original pathology reports and available hematoxylin and eosin-stained sections. Regional lymph node involvement was determined histopathologically. Tumors were staged according to the 8th edition of the American Joint Committee on Cancer (AJCC) melanoma staging system [28] (Figure 5).

### 4.2. Histopathologic Review and Tissue Selection

Archived hematoxylin and eosin-stained slides were reviewed by a pathologist with experience in dermatopathology, blinded to survival outcomes and immunohistochemical results. The original diagnosis, histologic subtype, Breslow thickness, ulceration, and other relevant histopathologic parameters were confirmed during this review.

For each case, the FFPE block containing the largest representative area of viable invasive melanoma was selected for immunohistochemical analysis. Areas of extensive necrosis, hemorrhage, cautery artifact, and insufficient viable tumor were avoided. Sections approximately 4 μm in thickness were prepared from the selected blocks and mounted on positively charged glass slides.

### 4.3. Immunohistochemistry and Evaluation

Immunohistochemical staining was performed on 4-μm sections from representative FFPE melanoma tissue blocks using an automated staining platform (Roche Ventana Benchmark Ultra, Basel, Switzerland). Following deparaffinization and heat-induced epitope retrieval, the sections were incubated with antibodies against PD-L1 (rabbit monoclonal antibody, SP263, 1:100 dilution, Roche Ventana, Oro Valley, AZ, USA), GPX4 (rabbit monoclonal antibody EPNCIR144; 1:500 dilution; Abcam, Cambridge, UK), and CD71 (mouse monoclonal antibody, MRQ-48; 1:100 dilution; Cell Marque, Rocklin, CA, USA). Immunoreactivity was visualized using 3,3′-diaminobenzidine and counterstained with hematoxylin.PDL1: tonsil; GPX4: human testis tissue; and CD71: Placental tissue positive control.

Stained sections were independently evaluated by pathologists (R.O.Y. and H.R.E.) blinded to the clinicopathologic and survival data. Interobserver agreement was excellent, with Cohen’s kappa coefficients of 0.92 for PD-L1, 0.90 for GPX4, and 0.92 for CD71. Discrepant cases were jointly reviewed by the two pathologists using a multiheaded microscope, and a final score was assigned by consensus. PD-L1 expression was assessed based on membranous staining in viable tumor cells and classified as positive when ≥1% of tumor cells were stained [29]. GPX4 expression was evaluated according to cytoplasmic staining, whereas CD71 expression was assessed based on membranous and/or cytoplasmic staining. GPX4 was classified as positive %5 and CD71 or negative using predefined cutoffs [30,31].

For combined biomarker analysis, cases positive for all three markers were classified as PD-L1^+^/GPX4^+^/CD71^+^, whereas triple-negative cases were classified as PD-L1^−^/GPX4^−^/CD71^−^. Survival outcomes were compared between these expression profiles.

### 4.4. Follow-Up and Survival Outcomes

Clinical follow-up data were obtained from institutional electronic medical records. The primary survival endpoints were overall survival (OS) and disease-free survival (DFS). OS was defined as the interval from the date of primary diagnosis to death from any cause or the date of the last documented follow-up for patients who remained alive. DFS was defined as the interval from the date of definitive surgery to the first documented local recurrence, regional recurrence, distant metastasis, or death.

The median follow-up duration was calculated from the date of diagnosis to the date of death or last clinical follow-up. Survival status and recurrence data were determined from clinical follow-up records.

### 4.5. Statistical Analysis

Statistical analyses were performed using IBM SPSS Statistics version 27 (IBM Corp., Armonk, NY, USA), with two-sided *p*-values <0.05 considered statistically significant. Categorical variables were summarized as frequencies and percentages, and continuous variables as mean ± standard deviation or median (interquartile range), as appropriate. Normality was assessed using the Shapiro–Wilk test. Associations between biomarker expression and clinicopathologic variables were evaluated using Pearson’s χ^2^ or Fisher’s exact test, as appropriate.

Survival probabilities for OS and DFS were estimated using the Kaplan–Meier method and compared using the log-rank test. Cox proportional hazards regression was used to calculate hazard ratios (HRs) with 95% confidence intervals (CIs). Variables with statistical significance or potential prognostic relevance in univariable analyses were considered for multivariable models. Given the limited number of events, parsimonious models with a restricted number of covariates were constructed to minimize the risk of overfitting, and the multivariable analyses were considered exploratory. For OS, Model 1 included lymphovascular invasion and GPX4, while Model 2 additionally included CD71. For DFS, Model 1 included lymphovascular invasion and GPX4, and Model 2 additionally included sex. The proportional hazards assumption was assessed using Schoenfeld residuals. No adjustment for multiple comparisons was performed because of the exploratory nature of the study.

## 5. Conclusions

GPX4 expression showed independent prognostic value for overall survival, whereas CD71 was associated with more invasive tumor features and poorer outcomes but did not retain independent significance in multivariable analysis. PD-L1 alone showed limited prognostic value. In contrast, the triple-positive phenotype was associated with significantly shorter overall and disease-free survival, although this finding should be interpreted cautiously given the exploratory nature of the analysis. Taken together, our findings indicate that integrating markers of immune escape, iron metabolism, and ferroptosis resistance may provide prognostic information beyond that obtained from individual biomarkers. Further validation in larger, independent cohorts is needed to determine the potential utility of this combined biomarker profile for risk stratification in cutaneous melanoma.

## Figures and Tables

**Table 1:** Demographic and clinicopathological characteristics of the study cohort.

Variable	Category	n	%
Age, years	<45	3	6.8
	≥45	41	93.2
Sex	Female	18	40.9
	Male	26	59.1
Comorbidity	Absent	7	15.9
	Present	37	84.1
Tumor location	Head/neck	11	25.0
	Trunk	4	9.1
	Upper extremity	12	27.3
	Lower extremity	17	38.6
Histologic type	Superficial spreading melanoma	21	47.7
	Nodular melanoma	18	41.0
	Lentigo maligna melanoma	5	11.3
Family history	Absent	39	88.6
	Present	5	11.4
Neurotropism	Absent	38	86.4
	Present	6	13.6
Perineural invasion (PNI)	Absent	36	81.8
	Present	8	18.2
Lymphovascular invasion (LVI)	Absent	32	72.7
	Present	12	27.3
Tumor-infiltrating lymphocytes (TILs)	Absent	33	75.0
	Present	11	25.0
Ulceration	Absent	25	56.8
	Present	19	43.2
Microsatellitosis	Absent	37	84.1
	Present	7	15.9
Breslow thickness	<1.0 mm	11	25.0
	>1.0–2.0 mm	4	9.1
	>2.0–4.0 mm	13	29.5
	>4.0 mm	16	36.4
Clark level	I	5	11.4
	II	8	18.2
	III	13	29.5
	IV	18	40.9
Stage	I	9	20.5
	II	15	34.1
	III	20	45.5
BRAF V600E mutation	Absent	36	81.8
	Present	8	18.2
Lymph node involvement	Absent	24	54.5
	Present	20	45.5

**Table 2:** Associations of PD-L1, CD71, and GPX4 expression with clinicopathological characteristics in patients with cutaneous melanoma.

Variable	Category	PD-L1 Neg. n (%)	PD-L1 Pos. n (%)	*p*	CD71 Neg. n (%)	CD71 Pos. n (%)	*p*	GPX4 Neg. n (%)	GPX4 Pos. n (%)	*p*
Age, years	<45	1 (3.0)	2 (18.2)	0.150	1 (3.6)	2 (12.5)	0.296	1 (3.7)	2 (11.8)	0.329
	≥45	32 (97.0)	9 (81.8)		27 (96.4)	14 (87.5)		26 (96.3)	15 (88.2)	
Sex	Female	14 (42.4)	4 (36.4)	0.504	14 (50.0)	4 (25.0)	0.095	14 (51.9)	4 (23.5)	0.060
	Male	19 (57.6)	7 (63.6)		14 (50.0)	12 (75.0)		13 (48.1)	13 (76.5)	
Comorbidity	Absent	2 (6.1)	5 (45.5)	0.007	3 (10.7)	4 (25.0)	0.205	3 (11.1)	4 (23.5)	0.248
	Present	31 (93.9)	6 (54.5)		25 (89.3)	12 (75.0)		24 (88.9)	13 (76.5)	
Family history	Absent	30 (90.9)	9 (81.8)	0.317	25 (89.3)	14 (87.5)	0.608	26 (96.3)	13 (76.5)	0.065
	Present	3 (9.1)	2 (18.2)		3 (10.7)	2 (12.5)		1 (3.7)	4 (23.5)	
Tumor location	Head/neck	6 (18.2)	5 (45.5)		8 (28.6)	3 (18.8)	0.397	8 (29.6)	3 (17.6)	0.706
	Trunk	4 (12.1)	0 (0)	0.281	4 (14.3)	0 (0.0)		3 (11.1)	1 (5.9)	
	Upper extremity	10 (30.3)	2 (18.2)		5 (17.9)	7 (43.8)		4 (14.8)	8 (47.1)	
	Lower extremity	13 (39.4)	4 (36.4)		11 (39.3)	6 (37.5)		12 (44.4)	5 (29.4)	
Neurotropism	Absent	28 (84.8)	10 (90.9)	0.527	26 (92.9)	12 (75.0)	0.116	24 (88.9)	14 (82.4)	0.426
	Present	5 (15.2)	1 (9.1)		2 (7.1)	4 (25.0)		3 (11.1)	3 (17.6)	
Histologic type	Superficial spreading	14 (42.4)	7 (63.6)	0.470	12 (42.9)	9 (56.3)	0.611	14 (51.9)	7 (41.2)	0.775
	Nodular	15 (45.5)	3 (27.3)		13 (46.4)	5 (31.3)		10 (37.0)	8 (47.1)	
	Lentigo maligna	4 (12.1)	1 (9.1)		3 (10.7)	2 (12.5)		3 (11.1)	2 (11.8)	
PNI	Absent	29 (87.9)	7 (63.6)	0.092	26 (92.9)	10 (62.5)	0.019	24 (88.9)	12 (70.6)	0.130
	Present	4 (12.1)	4 (36.4)		2 (7.1)	6 (37.5)		3 (11.1)	5 (29.4)	
LVI	Absent	25 (75.8)	7 (63.6)	0.339	23 (82.1)	9 (56.3)	0.068	22 (81.5)	10 (58.8)	0.098
	Present	8 (24.2)	4 (36.4)		5 (17.9)	7 (43.8)		5 (18.5)	7 (41.2)	
Ulceration	Absent	18 (54.5)	7 (63.6)	0.434	16 (57.1)	9 (56.3)	0.600	15 (55.6)	10 (58.8)	0.541
	Present	15 (45.5)	4 (36.4)		12 (42.9)	7 (43.8)		12 (44.4)	7 (41.2)	
Microsatellitosis	Absent	28 (84.8)	9 (81.8)	0.571	25 (89.3)	12 (75.0)	0.205	24 (88.9)	13 (76.5)	0.248
	Present	5 (15.2)	2 (18.2)		3 (10.7)	4 (25.0)		3 (11.1)	4 (23.5)	
TILs	Absent	27 (81.8)	6 (54.5)	0.083	22 (78.6)	11 (68.8)	0.354	20 (74.1)	13 (76.5)	0.576
	Present	6 (18.2)	5 (45.5)		6 (21.4)	5 (31.3)		7 (25.9)	4 (23.5)	
BRAF V600E	Absent	26 (78.8)	10 (90.9)	0.343	23 (82.1)	13 (81.3)	0.620	22 (81.5)	14 (82.4)	0.635
	Present	7 (21.2)	1 (9.1)		5 (17.9)	3 (18.8)		5 (18.5)	3 (17.6)	
Breslow thickness	<1.0 mm	9 (27.3)	2 (18.2)	0.004	10 (35.7)	1 (6.3)	0.140	9 (33.3)	2 (11.8)	0.209
	>1.0–2.0 mm	0 (0.0)	4 (36.4)		1 (3.6)	3 (18.8)		2 (7.4)	2 (11.8)	
	>2.0–4.0 mm	11 (33.3)	2 (18.2)		8 (28.6)	5 (31.3)		7 (25.9)	6 (35.3)	
	>4.0 mm	13 (39.4)	3 (27.3)		9 (32.1)	7 (43.8)		9 (33.3)	7 (41.2)	
Clark level	I	3 (9.1)	2 (18.2)	0.501	5 (17.9)	0 (0.0)	0.034	5 (18.5)	0 (0.0)	0.231
	II	7 (21.2)	1 (9.1)		6 (21.4)	2 (12.5)		5 (18.5)	3 (17.6)	
	III	8 (24.2)	5 (45.5)		8 (28.6)	5 (31.3)		6 (22.2)	7 (41.2)	
	IV	15 (45.5)	3 (27.3)		9 (32.1)	9 (56.3)		11 (40.7)	7 (41.2)	
Stage	I	7 (21.2)	2 (18.2)	0.578	9 (32.1)	0 (0.0)	0.045	9 (33.3)	0 (0.0)	0.137
	II	12 (36.4)	3 (27.3)		8 (28.6)	7 (43.8)		6 (22.2)	9 (52.9)	
	III	14 (42.4)	6 (54.5)		11 (39.3)	9 (56.3)		12 (44.4)	8 (47.1)	
Lymph node involvement	Absent	19 (57.6)	5 (45.5)	0.362	17 (60.7)	7 (43.8)	0.220	15 (55.6)	9 (52.9)	0.555
	Present	14 (42.4)	6 (54.5)		11 (39.3)	9 (56.3)		12 (44.4)	8 (47.1)	

**Table 3:** Univariable Cox regression analyses for overall survival and disease-free survival.

Variable	Category/Reference	OS HR (95% CI)	*p*	DFS HR (95% CI)	*p*
Age group	<45 years (Ref.)	Reference	—	Reference	—
	≥45 years	1.353 (0.172–10.623)	0.773	1.427 (0.183–11.119)	0.734
Sex	Female (Ref.)	Reference	—	Reference	—
	Male	3.957 (0.854–18.337)	0.079	4.495 (0.985–20.510)	0.052
Tumor localization	Head/neck (Ref.)	Reference	—	Reference	—
	Trunk	1.145 (0.103–12.775)	0.912	1.183 (0.106–13.194)	0.892
	Upper extremity	2.929 (0.526–16.315)	0.220	2.725 (0.489–15.173)	0.253
	Lower extremity	2.392 (0.443–12.923)	0.311	3.046 (0.592–15.660)	0.182
Ulceration	Absent (Ref.)	Reference	—	Reference	—
	Present	1.502 (0.473–4.767)	0.490	1.856 (0.602–5.719)	0.282
Neurotropism	Absent (Ref.)	Reference	—	Reference	—
	Present	1.604 (0.416–6.186)	0.493	1.425 (0.378–5.372)	0.601
Histologic type	Superficial spreading (Ref.)	Reference	—	Reference	—
	Nodular	1.502 (0.334–5.110)	0.323	1.888 (0.891–7.558)	0.098
	Lentigo maligna	1.302 (0.122–5.006)	0.615	1.657 (0.435–6.120)	0.246
PNI	Absent (Ref.)	Reference	—	Reference	—
	Present	2.101 (0.631–6.998)	0.227	1.836 (0.564–5.973)	0.313
LVI	Absent (Ref.)	Reference	—	Reference	—
	Present	3.302 (1.058–10.305)	0.040	4.086 (1.368–12.201)	0.012
Microsatellitosis	Absent (Ref.)	Reference	—	Reference	—
	Present	2.159 (0.631–7.386)	0.220	1.892 (0.568–6.300)	0.299
BRAF V600E mutation	Negative (Ref.)	Reference	—	Reference	—
	Positive	0.879 (0.192–4.024)	0.868	1.386 (0.380–5.049)	0.621
Breslow thickness	Per category increase	1.236 (0.710–2.154)	0.454	1.475 (0.827–2.631)	0.188
Clark level	Per level increase	0.825 (0.456–1.493)	0.525	0.782 (0.451–1.355)	0.380
Lymph node involvement	Negative (Ref.)	Reference	—	Reference	—
	Positive	0.926 (0.291–2.946)	0.896	1.624 (0.495–5.327)	0.424
TILs	Absent (Ref.)	Reference	—	Reference	—
	Present	1.534 (0.459–5.126)	0.487	1.308 (0.401–4.264)	0.657
Stage	Stage III (Ref.)	Reference	—	Reference	—
	Stage I	0.918 (0.190–4.448)	0.916	0.646 (0.138–3.020)	0.579
	Stage II	0.476 (0.098–2.313)	0.357	0.170 (0.021–1.355)	0.094
PD-L1 expression	Negative (Ref.)	Reference	—	Reference	—
	Positive	2.139 (0.675–6.774)	0.196	1.819 (0.592–5.590)	0.296
CD71 expression	Negative (Ref.)	Reference	—	Reference	—
	Positive	3.178 (0.996–10.138)	0.051	1.813 (0.602–5.460)	0.290
GPX4 expression	Negative (Ref.)	Reference	—	Reference	—
	Positive	5.781 (1.559–21.434)	0.009	4.221 (1.295–13.760)	0.017

HR, hazard ratio; CI, confidence interval; OS, overall survival; DFS, disease-free survival; PNI, perineural invasion; LVI, lymphovascular invasion; TILs, tumor-infiltrating lymphocytes.

**Table 4:** Multivariable Cox regression analyses for overall survival and disease-free survival.

Variable	OS Model 1 HR (95% CI)	*p*	OS Model 2 HR (95% CI)	*p*	DFS Model 1 HR (95% CI)	*p*	DFS Model 2 HR (95% CI)	*p*
LVI	2.402 (0.739–7.809)	0.145	2.139 (0.580–7.890)	0.254	3.172 (1.025–9.810)	0.045	3.911 (1.174–13.031)	0.026
GPX4	4.845 (1.278–18.369)	0.020	4.333 (1.022–18.368)	0.047	3.335 (0.997–11.164)	0.051	1.780 (0.433–7.313)	0.424
CD71	—	—	1.337 (0.330–5.420)	0.684	—	—	—	—
Sex	—	—	—	—	—	—	3.678 (0.640–21.150)	0.145

HR, hazard ratio; CI, confidence interval; OS, overall survival; DFS, disease-free survival; LVI, lymphovascular invasion.

## Data Availability

The datasets used in this study can be made available by the corresponding author upon reasonable request, with permission from the Dermatology Department of Sivas Cumhuriyet University School of Medicine.

## Abbreviations

AJCC	American Joint Committee on Cancer
BRAF	B-Raf proto-oncogene
CD71	Transferrin receptor 1
CI	Confidence interval
DFS	Disease-free survival
FFPE	Formalin-fixed paraffin-embedded
GPX4	Glutathione peroxidase 4
HR	Hazard ratio
LVI	Lymphovascular invasion
OS	Overall survival
PD-1	Programmed death-1
PD-L1	Programmed death-ligand 1
PNI	Perineural invasion
TILs	Tumor-infiltrating lymphocytes
